# Potential for establishing an injury surveillance system in India: a review of data sources and reporting systems

**DOI:** 10.1186/s12889-020-09992-9

**Published:** 2020-12-14

**Authors:** Jagnoor Jagnoor, Manickam Ponnaiah, Matthew Varghese, Rebecca Ivers, Rajesh Kumar, Shankar Prinja, Aliki Christou, Tanu Jain

**Affiliations:** 1 Injury Division, The George Institute for Global Health, New Delhi, India; 2 University of New South Wales, Sydney, Australia; 3 National Institute of Epidemiology, Chennai, India; 4 St. Stephen’s Hospital, Delhi, India; 5 School of Public Health and Community Medicine, The University of New South Wales, Sydney, Australia; 6 School of Public Health, Post Graduate Institute of Medical Education and Research, Chandigarh, India; 7 School of Public Health, The University of Sydney, Sydney, Australia; 8 Directorate General of Health Services, New Delhi, India

**Keywords:** Injury surveillance, Unintentional injuries, Road traffic injuries, Drowning, Health systems, Epidemiology, India, Mortality, Morbidity

## Abstract

**Background:**

Unintentional injuries account for 10% of deaths worldwide; the majority due to road traffic injuries, falls, drowning, poisoning and burns. Effective surveillance systems provide evidence for informed injury prevention and treatment and improve recovery outcomes. Our objectives were to review existing sources of unintentional injury data, and quality of the data on the burden, distribution, risk factors and trends of unintentional injuries in India and to describe strengths and limitations of health facility-based data for potential use in injury surveillance systems.

**Methods:**

We searched national and international organisations’ websites to identify unintentional injury-related mortality and morbidity data sources in India. We reviewed and evaluated data collection methods for surveillance attributes recommended by World Health Organization (WHO). We visited health facilities at all levels from public and private sectors, emergency transport centres, insurance offices and police stations in settings reporting significant number of injuries. In these sites, we interviewed key stakeholders using an explorative approach on current data collection processes and challenges to establishing an injury surveillance system based on WHO guidelines.

**Results:**

Major gaps were highlighted in injury mortality and morbidity data in India, including ill-defined causes of injury deaths and lack of standardisation in classification and coding. Site visits revealed that reporting standards of injuries varied, with issues around clarity of definitions, accountability, time points and lack of reporter/coder training. Major challenges were lack of dedicated staff and training.

**Conclusions:**

There is an important need to build human resource capacity, integrate data sources, standardise and streamline data collected, ensure accountability and capitalise on digital health information systems including insurance databases.

**Supplementary Information:**

The online version contains supplementary material available at 10.1186/s12889-020-09992-9.

## Background

The World Health Organization (WHO) and the Global Burden of Diseases Study (GBD) 2017 estimate that there are 3.1 million deaths due to unintentional injuries worldwide [1]. Most of these deaths are attributable to road traffic injuries, falls, drowning, poisoning and burns.

In 2017, the GBD estimated that in India 977,117 deaths and 49,657,335 Disability-Adjusted Life Years (DALYs) were the result of injuries [1]. In 2015, the National Crime Records Bureau annual report, using data from police records, reported only 148,707 transport injury deaths, whereas the GBD in the same year estimated 243,089 deaths (using statistical modelling to estimate gaps in data) [1, 2]. Other sources of mortality data from selected rural health centres and urban hospitals are also not representative of the population of India and have other methodological limitations. From analysis of the nationally representative Million Death Study, 2010–2014 Menon et al. reported 676, 000 unintentional injury deaths, finding that road traffic injuries (275,000 deaths; 41% of unintentional injury deaths), falls (190,000 deaths, 28%) and drowning (62,000 deaths, 9%) were the three leading causes of unintentional injury mortality [3]. Unintentional injury death data direct from police data sources are likely to be grossly underreported and subject to misclassification.

The Ministry of Health and Family Welfare, India has been making efforts under the Trauma Programme to establish a national injury surveillance system with the motivation of to move towards health promotion and injury prevention, to reduce the health burden and for economic and social benefits.

There are three policy statements that demonstrate government investment in reducing unintentional injuries. These include India’s commitment to SDG Goal 3.6 to reduce road injuries by 50%, signatory to Stockholm declaration, 2020 by Ministry of Road, Transport and Highways and The National Trauma programme by Ministry of Health and Family Welfare. The new National Road Safety Strategy, currently being finalized, also envisages halving the number of road accident fatalities by 2025 [4].

Surveillance is the foundation of injury prevention and control, facilitating identification of emerging issues and high-risk populations and quantifying issues related to injury disparities in vulnerable populations. It is key to tracking trends, informing programme design, and aiding redesign, evaluation and development of more targeted and effective interventions. The lack of reliable data on the burden of injuries associated with hospitalisation, disability, functioning, quality of life, use of health services and cost, explains in part why there are gaps in investment for injury prevention and trauma care.

Generating reliable data has become more pressing for India in light of recent traction in reducing global road traffic injuries with the Sustainable Development Goals, as well as international calls from the 72nd World Health Assembly for the provision of emergency care [5]. A robust surveillance system will enable monitoring of some of the initiatives under Motor Vehicles Act, 2019 [6], such as cashless insurance for emergency trauma care services. Likewise, National Programme for Health Care of the Elderly [7] including fall prevention, and National Programme for Prevention and Management of Burn Injuries [8], are opportunities to build a surveillance system that provides robust data for guiding evidence-based interventions and an effective national trauma care system.

## Objectives

1. To review existing quality of data on on burden, distribution, risk factors and trends for unintentional injuries in India.
2. To describe the strengths and limitations of health facility-based data for potential use in injury surveillance systems.

## Methods

### Grey literature search

In May to June 2019, a desk review was conducted exploring available grey literature on unintentional injury burden, distribution, and risk factors in India. We searched the websites of national and international organisations to identify data sources for injury-related mortality and morbidity in India (Table 1). Primary data engines searched were Government of India portals and WHO- Iris. Most recent documents published between 2008 and 2018, reporting data on reported data on road traffic crashes or injuries and/or deaths and/or disability and/or risk factors were included. Opinion pieces or commentaries were excluded.

**Table 1:** Websites searched for grey literature on data sources related to injuries in India

National Data Sources	International data Sources
□ Vital registration records □ Sample registration system data □ District level health survey data/National Sample Survey Organisation data □ Reports from National Crime Records Bureau □ Reports from Ministry of Road Transportation and Highways □ Central Bureau of Health Intelligence □ Ministry of Health and Family Welfare □ Ministry’s and Departments such as Social Justice (disability and rehabilitation) □ Ministry of Environment, Forest and Climate Change □ Ministry of Labour and Employment □ Ministry of Statistics and Programme Implementation □ Integrated Disease Surveillance Programme □ Central Bureau for Health Intelligence □ Open data platforms □ Cab/taxi websites □ Insurance websites	Organisations: □ WHO □ World Bank □ UNICEF Networks: □ Road traffic Injury Research Networks □ Global Road Safety Partnerships

We reviewed the data collection methods used for each of the identified data source, reporting on their strengths and limitations. The data sources were independently evaluated by two researchers (JJ and AC) against the seven attributes of simplicity, flexibility, acceptability, reliability, utility, sustainability and timeliness, recommended by the WHO Injury Surveillance Guidelines [9].

### Settings

The selection of evaluation sites was informed by ranking of cause of injury deaths as reported by the National Crime Record Bureau [2], known high risk areas for specific injuries, for example drowning in the north east [10], geographic representation, and collaborative links using a purposive convenience sampling method. We visited sub-centres (which do not attend to injuries), primary healthcare centres, district hospitals, tertiary care centres, private and charitable hospitals, ambulance services, police stations, and insurance offices (Table 2).

**Table 2:** Evaluation sites visited January–March 2017

Site Number	Site Name
Site 1a	Postgraduate Institute of Medical Education and Research (PGIMER), Chandigarh
Site 1b	Police Post (PGIMER, Chandigarh), Chandigarh
Site 1c	PGIMER, Chandigarh Accident cell (Sector 23, Chandigarh), Chandigarh
Site 2a	District Hospital Fatehgarh Sahib, Punjab Community Health Centre, KHERA, Punjab Community Health Centre, Bassi Pathana, Punjab
Site 2b	
Site 2c	
Site 3a	Indira Gandhi Hospital, Shimla Himachal Pradesh PHC HP Secretariat, Shimla-2, Himachal Pradesh DDU, Zonal hospital, Himachal Pradesh
Site 3b	
Site 3c	
Site 4a	Sheth Vadilal Sarabhai General Municipal Hospital (SVSGH), Ahmedabad, Gujarat
Site 4b	Adalaj Community Health Centre, Adalaj, Gandhinagar, Gujarat
Site 4c	Primary Health Centre, Sughad, Gandhinagar, Gujarat
Site 4d	Civil hospital, Gandhinagar, Gujarat
Site 5	Charitable Hospital in Tezpur, Assam
Site 6a	Primary Health Centre (Kot, Haryana)
Site 6b	Community Health Centre, Raipur Rani, Haryana
Site 6c	Burns Unit: Department of Health Services, Haryana
Site 6d	Private Hospital (Panchkula, Haryana)
Site 7a	King George Medical University (Tertiary Care Centre), Uttar Pradesh
Site 7a	Police post (on KGMU campus), Uttar Pradesh
Site 7c	District Hospital, Barabanki, Uttar Pradesh
Site 7d	CHC, Badagaon Village (Primary health care), Uttar Pradesh
Site 8a	Bhartiya Vidyapeeth, Pune, Maharashtra
Site 9a	Primary Health Centre, Telengana
Site 9b	CHC/DH, Telengana
Site 9c	Tertiary Centre, NIMS, Telengana
Site 9d	Tertiary Centre, Ghandi hospital, Telengana
Site 9e	Police Outpost, Telengana
Site 10	Choithram Hospital and Research Centre, Indore, Madhya Pradesh
Site 11	CMC, Vellore, Tamil Nadu

Ethics for the research was approved by the Postgraduate Institute of Medical Education and Research Chandigarh (PGIMER) and informed written consent was obtained from all participants.

### Site visits

We adapted an online data collection form from the WHO injury surveillance evaluation tool using Research Electronic Data Capture (REDCap) [9]. We found that data collection on a closed structure form limited our capacity to identify inefficiencies and to explore bigger issues around resources, administration and reporting. Health facilities had no directives/guidelines to be accountable for with respect to injury data. We therefore took an explorative approach and identified details on the population, time periods, and type of information collected (see Additional file 1 for adapted tool). We also explored storage, use, transfer, analysis and reporting of data.

In- depth interviews were conducted (*n* = 28) using purposive sampling with a range of relevant personnel such as emergency registration clerks, information assistants, nurses, medical officers, civil surgeons, medical record officers, ambulance drivers, attendants, call centre staff, insurance officers and police officials involved in record keeping at any stage of injury/ trauma care. Validated survey forms, collecting information on process evaluation and system environment evaluation tool was used [11]. Interviews were part of the site evaluations.

In January to March 2017, we interviewed hospital staff attending injured person at the time of entry to the health facility and person taking patient history, as well as medical record keeping and reporting staff. Interviews were conducted with staff from ambulance and call centres under the National Highway Accident Relief Service Scheme, and insurance company representatives were also approached. We visited 6 police stations in Chandigarh, Uttar Pradesh, Rajasthan and those in Delhi to Jaipur route.

## Results

### Grey literature search

Fifteen online sources contributed evidence to this review (Supplementary Table 1). National-level data were provided by the National Crime Records Bureau from police registration of injuries and deaths [2]. This data was also used by the Ministry of Road Transport and Highways [12], the Ministry of Statistics and Programme Implementation [13], and the Open Government Data Platform [14]. A key primary national data source was the Medical Certification of Cause of Death held by the Registrar General of India [15]. Medical Certification of Cause of Death is also the main data source used to extrapolate and estimate figures for the GBD, also included in this review [1]. The findings highlight the limitations with respect to inherent biases in reporting based on single data source such as hospital-based data or police-based data and thus lack of representativeness of the data. However, potential of multiple data source linkage is also limited as databases are not comparable with lack of standardization in definition, coding and reporting.

Three websites reported data from community surveys, two at the national level. The National Sample Survey Office (now known as National Statistical Office), also under the Ministry of Statistics and Programme Implementation [13], collected self-reported data on hospitalisations as well as reporting cost and use of facilities. The Ministry of Home Affairs collected cause of death statistics from nationally representative household census surveys [16]. Both sources provide national data every 10 years. Young Lives is a prospective longitudinal qualitative study based in Andhra Pradesh with surveys repeated every 4 years however we were unable to access the most recent data for injury burden [17].

Hospital-based data were obtained from a 1 month pilot report from the National Injury Surveillance Trauma Registry and Capacity Building Centre [18] (New Delhi only). The Integrated Disease Surveillance Programme [19] run by the Ministry of Health and Family Welfare provides weekly hospital-based reports for all of India (but only two injury categories are included; dog and snake bite). The Ministry of Labour and Employment reported data annually on injuries occurring in mines [20]. The Ministry of Environment, Forest and Climate Change was identified as a potential source to provide drowning injury data in the future although none was available in the current report [21].

Two reports from the WHO were included, one on road safety [22] using data extrapolated from the Ministry of Road Transport and Highways [12], and a report on drowning [23] which had no national-level data available.

The sources were all assessed and rated according to attributes of injury surveillance guidelines and utility of data for surveillance (Supplementary Table 2).

### Findings from site visits document review and in- depth interviews

Healthcare providers at different levels of the healthcare system are a source of data regarding outcomes of injury events. Hospital discharge records are also important sources of injury morbidity data as they include injuries severe enough to warrant hospitalisation and report on the discharge outcome of the injured person (Table 3).

**Table 3:** Summarized notes from interview with stakeholders

Recommendations based on desk review	Recommendations based on data source evaluation
Integrated Disease Surveillance Programme (IDSP): IDSP has been able to collate weekly disease surveillance data through IDSP portal from 91% of the districts in the country; data on epidemic-prone diseases are being collected from reporting units such as sub-centres, primary health centres, and community health centres, hospitals including government and private sector hospitals and medical colleges. At the moment two types of injuries are collected under the systems namely “dog bite” and “snake bite”. A piggy back of 3–4 key variables to guide state led interventions may be considered. This would include key identifiers, mechanism of injury and mode of transportation to the health facility. Additionally based on stakeholder capacity “I Form” for details injury information may be piloted in some districts for each state.	Standardize coding systems: Utilize common definitions of injury and a coding system that is transferable across the data sources.
Sample Registration System: Data from Sample Registration System is ideal for looking into trends for all unintentional injuries mortality – a representative sample with high validity for identifying cause of injury deaths. Whilst the data is inadequate to guide interventions it provides robust data on regional and national injury burden, helping identify high risk populations.	Police data source improvements: Police have the capacity to include pre-event and at the time of injury events in their dataset; some of these are covered for road injuries through the tools developed by the Transport Research Wing, however quality of data varies and also further work is needed to develop tools relevant for each type of injury.
Civil Registration System: Proportionate mortality data can also be explored through Civil Registration System in states like Maharashtra, Punjab and like with high coverage can also be explored. Strengthening through “Form 4b- Lay Man” reporting of death certification. Injury deaths are unlikely to be misclassified, reporting on cause of death by next of kin has good validity.	Single unique patient identifiers: or where available ADHAR card number, the date of injury, date of birth, gender- as applicable National Identification Number (NIN) for health facilities to generate a UID. Essentially a common Health management Information System would have an overarching benefit across all disease surveillance systems such RNTCP (Revised national tobacco control programme), NACO (National AIDS control organisation) or in cases of an epidemic or an outbreak of communicable diseases, IDSP.
Special surveys: Technical experts for injuries should be consulted in development of special surveys such as District Level Health Survey (DLHS), National Sample Survey Office (NSSO) and National Family Health Survey (NFHS); this would assist in mitigating limitations such as lack the standardisation of question which inhibits data utilisation	Injury severity coding: Whilst there is clearly a need for strengthening ICD training and coding, injury morbidity data is also essential for evaluating trauma services. Injury severity coding such as AIS, ISS or NISS should be incorporated at district and tertiary care centres.
Police or Transport data: Reporting bias in police data is difficult to address, however for the purpose of reporting injuries it would be beneficial to include all First Information Reports (FIR)/ cases in daily dairies and not only cases registered under Indian Penal Code, so as to report a more representative data in the annual report.	Use of Insurance data: Insurance data is extensive and uses multiple informants using police, vehicle registration, hospital, injured person and cost data. However it is important to ensure completeness, standardisation of data, and reporting of data are essential to guide future health and social insurance initiatives.
Research from academic institutes: Several project work related to injuries are taken up at post-graduate medical and research institutes in India. Most of them report on retrospective medical record review, with methodological limitations. Research using prospective longitudinal methods should be encouraged.	Major trauma registrations: Injuries resulting in major trauma such as traumatic brain injury, spinal cord injury, burns, amputations and other impairments require rehabilitation, a registry at major trauma centre following on recovery post discharge should be explored.

#### Public health system

We evaluated a total of six Primary Health Centres and 14 secondary-level health facilities (Community Health Centres and District Hospitals). We observed that resources are available to reliably collect information on injuries. The systems are weak at Primary Health Centres; however, each employs an information assistant or pharmacist, who has computer skills and is capable of being trained in International Classification of Diseases (ICD) coding. The reporting at Primary Health Centres and Community Health Centres varied from printed registers to handwritten notes in a notebook, with variables such as age, sex, date, site and mechanism of injury in a few words.

The Community Health Centres and District Hospitals across all states functioned well with respect to recording and reporting and coding data as per the given reporting form. However, there was less clarity around what was being recorded (with respect to definitions) and at what time points, once again reflecting a lack of standardisation.

Health facilities report on number of trauma presentations, inpatient and outpatient department admissions, and minor and major surgeries on weekly, monthly and quarterly bases. Of 210,704 health facilities in the public sector, 202,123 (96%) reported monthly data for the previous 12 months [24]. District Hospitals generate three monthly reports. It includes, the *health management information report* that has number of admitted medico-legal cases. The majority of District Hospitals were using a Health Management Information System and thus having potential for data linkages. Another report generated by district hospital is that of *Compiled report of hospitals at the sub-district level* with some information on how ICD coding is used, as new classifications compile several ICD codes when reporting on the number of cases, out-patients, in-patients and deaths. The ICD codes are grouped, and new 3-digit numerical codes introduced. These might suffice for administrative purposes, reporting on how and what broad conditions require use of health facilities, however, the information is very limited for guiding public health interventions. Additionally, 30–60% had ‘garbage’ codes, i.e. “other”, across sites. Third type of report generated from district hospital is the *Monthly report on recorded non-communicable diseases.* The report lost further details in coding, with all injuries accumulated under “Accident injuries”. The report is sent to State zonal offices representing the Central Bureau of Health Intelligence. Only three rows were used for injury cases, reporting male/female distributions of inpatient, outpatients and deaths for road traffic injuries, snake bites and other injuries. The annual national report by the Central Bureau of Health Intelligence does not report injury statistics. In the public system, staff involved in coding and reporting data were not trained and used an abridged coding guide (all injuries were coded as either transport accidents or all other accidents) with limited understanding of the underlying definitions and classifications.

#### Other health facilities

Tertiary health facilities used a robust ICD coding system reporting 3-digit codes using both Chapter XIX and Chapter XX, and staff had undergone ICD training. However, the codes are not compiled or reported to any authority even at the hospital administration level, so data remain in the Medical Records Department. Even the smaller charitable hospitals reported having trained ICD coders; coding was not seen at private hospital sites. While tertiary-level sites reported receiving ICD coding training, refresher training has not been done for years and often new staff are trained by their peers.

#### Pre-hospital data on injuries

Pre-hospital data recorded by ambulance services or the associated call centre is patchy (Supplementary Table 3). For most sites, the associated ambulance service did not collect information on injury or the status of the injured. It was observed that good infrastructure existed, but there was a gap in personnel training. Reporting was reliable for process variables including dispatch time, place of pick-up, pick-up time and admission time. Only two sites recorded injury/trauma details, both of which had a pilot injury/trauma system either previously or currently funded through a grant. At most sites, ambulances were infrequently used to transport trauma patients, ranging from 1 to 30% of trauma cases at most.

#### Examples of challenges and opportunities in documenting specific injuries

In the North Eastern India, frequency of drowning is high, particularly among children under 5 years [25]. In stakeholder interviews in Assam and West Bengal, accredited social health activist (ASHA) trainers highlighted that deaths in young children are prone to underreporting, due to limited birth registrations. Further, fatal drownings rarely present to a health facility, and health facilities can at best capture non-fatal drownings leading to impairments. The burden of drowning is best captured through community-based surveys and surveillance systems.

For burns, all public health facilities had a specific form for recording in-depth information on patient history and diagnosis. The information was collected and recorded by either the general surgeon or a plastic surgeon and appeared to be detailed and complete at all sites. However, this data was not used or collated at the hospital record department, thus leading to duplication of reporting efforts. Inadequacies of emergency departments to manage burns care and the need for additional units were emphasised, and the plans for burns units and their progress, under the National *Programme* on Prevention and Management of *Burn* Injuries [7, 8] were a welcome step. Private and charitable facilities had better post-discharge follow-up and rehabilitation records.

Some hospital administrators referred to the National Accreditation Board for Hospitals and Healthcare Providers and proposed that surveillance systems for all conditions could be strengthened through the Board’s guidebook.

A parallel reporting system is that of medico-legal cases. The form used for medico-legal cases was standardised across sites, although case definition/reporting varied. At some sites, all injury cases were reported to the police and the form filled for all trauma records, while at other facilities it was up to the injured person/ family or the treating physician to decide if the injury constituted a medico-legal case. Deaths, particularly “unnatural deaths”, which included injuries, food poisoning, pregnancy-related, and sudden heart attack were almost always reported as medico-legal cases.

### Other data sources

The National Crime Records Bureau, under the Ministry of Home Affairs, collects, collates and analyses statistics on injury incidents and deaths due to accidents and suicides reported to the police. It has published annual reports titled “Accidental Deaths and Suicides in India”, since 1976. The published data broadly classifies injury into accidents (unintentional injury deaths) and suicides.

Further, we investigated availability and remit of insurance data as source of injury surveillance indicators. The representatives from insurance companies associated with the National Highway Accident Relief Service Scheme reported that all the hospitals in the scheme were provided with the same health management information system to enable uniformity in data collection. Road trauma specific cashless insurance schemes and Universal health coverage delivered insurance are in nascent stages of their implementation. This is a timely opportunity to develop and implement systems of transparency and accountability through good data for injury surveillance and management.

At hospital sites, copies of all information recorded and reported to insurance companies was maintained, although there was little clarity on how reports were generated for cause of injury. We explored Insurance Regulatory and Development Authority of India annual reports, but no relevant information was found. Guidelines for standardisation of health insurance were reviewed and use of definitions comparable with health sector definitions for injury would be advisable to enable better use of data. Insurance data also has limited coverage; however, data is completely valid, and has the potential to report cost, injury morbidity and rehabilitation care across road injuries and occupational injuries such as electrical and chemical burns.

## Discussion

Based on the review of injury data sources and systems along with inspection of few relevant sites and settings and interviewed key stakeholders in India, we identified data gaps and recognized the need for a standardised, nationally coordinated approach to injury surveillance. In recent times, some investment is being made in trauma registry however implementation on injury surveillance remains challenging and some of the attributing factors are a) infectious conditions, maternal and child health are high priority for program implementation and monitoring; injury only moving up the agenda with recent global mandates like Decade for Road Safety Action and SDG’s; b) resources and coordination are required for good data and the system may lack capacity as other competing priorities exist; c) health systems pre- dominantly manages injuries and thus can inform trauma control interventions. However, to inform injury prevention programs and policies, multisectoral collaboration is required.

We identified several gaps in injury mortality data. Firstly, a significant proportion of injury deaths had unspecified and ill-defined causes in the available data sources. Secondly, each of the reviewed data sources used modified versions of the ICD-10 coding system and often poorly categorised external causes of injuries. Thirdly, we identified lack of standardization for the categorisation and stratification of data (for example, by age) across the data sources and therefore limiting comparability of data. Finally, we identified that deaths are reported as a proportion of all-cause mortality. Proportionate mortality does not help in reporting actual magnitude of the burden since it is a numerator-based indicator. Thus, denominator data and standardisation are essential, which are often not available in the Systematic Country Diagnostic (rural) and the Medical Certification of Cause of Death (urban) reports. The verbal autopsy-based Sample Registration System cause of death is a reliable data source for measuring injury burden. However, such sample-based data provides mortality burden at the National/ State level with very limited insight on contextual risk factors, and this not ideal for guiding injury reduction interventions.

With reference to injury morbidity and its risk factors, we identified that data describing this is very limited. Additionally, available data suffer from non-standardized use of definitions, classification and reporting methods. We could not identify any reliable National level data source for injury-associated morbidity.

Among the data sources identified, the Open Government Data Platform under the National Data Sharing and Accessibility Policy has the potential to be a good resource for injury surveillance data. In a federal set up much depends on efforts of States to mobilise data on road injuries and health. Population based bottom -up systems like Integrated Disease Surveillance Programme [24] hold immense potential to strengthen injury surveillance data but us under-funded and lack the human resource capacity. However, the limitations of the primary data sources, as pointed out above, need to be addressed. We identified some pilot initiatives as part of the Global Burns Registry and National Injury Surveillance Trauma Registry. Between June 2019 and 2020, the work undertaken has informed the Minimum Data set, injury surveillance format, standard operating procedures for data entry for National Injury Surveillance Centre, which is currently being implemented across 14 states and 46 hospital sites [26]. These initiatives have the potential to provide robust timely data on risk factors and acute care. However, they are prone to typical biases linked to hospital-based data, such as the invisibility of minor injuries, those which result in death at the scene as well as groups who cannot access hospital treatment for financial or geographical reasons. Therefore, these initiatives need to be supplemented by population-based or multi-source data.

The published annual data from National Crime Records Bureau does not permit us to compare with International Classification of Disease due to variation in coding categories and therefore classification. Further, the data in these reports are gross underestimates for several known reasons [27] such as lack of awareness about the procedure, requirements for completing first information reports and the drawn-out administrative processes including required court attendance. The stigma associated with injuries like suicides [28] and burns can be a deterrent and there is a lack of motivation to report falls, accidental poisonings and drownings as they are not considered to have any legal implications.

As observed in hospital settings, reporting of medico-legal case injuries is also variable. Although the published literature emphasises the issues related to reporting bias [29] there is the possibility of undercount due to the logistics of preparing the National Crime Records Bureau annual reports, especially for injury-related morbidity. Daily diaries and first information reports from police stations are another source of information. However, they do not get counted until case registration under Indian Penal Code. Therefore, the data are not reflected in any report beyond the station. In fact, according our observations, nearly half of the cases get excluded from police statistics.

Overall, the National Crime Records Bureau has the potential to be an excellent source of data for pre-injury and crash data, providing robust evidence on risk factors associated with injury incidents despite known undercounts. The Ministry of Road Transport and Highways’ Transport Research Wing extracts information (based on a 19-item format) from police data for their annual report on road traffic injuries with detailed information on context of road injury.

Extrapolated global data sources like the GBD study are good for advocacy but provide little information to guide the development of local-context specific evidence-based interventions to reduce the burden associated with injuries. We identified a paucity of information on hospital discharge, recovery outcomes and rehabilitation in the reviewed data sources. Another important stakeholder and data source are that of the insurance sector. However, no data from the insurance sector is available in the public domain. Insurance-based cost data on injuries can help build an economic case for injury prevention and control so it is recommended that access to such data be facilitated.

To establish an effective injury surveillance system in India, the definitions and methods of coding and reporting in the existing data sources require standardisation. The system should integrate insurance cost data and other available data sources. Dedicated personnel need to be appointed to facilitate robust monitoring and accountability of the system.

Our study had several limitations. Our review was restricted to major data sources that reported aggregated data. We used search strategies sensitive enough to identify all major data sources from grey literature. Hence, it is likely that we have not missed any such data sources. Nevertheless, any future work needs consider reviewing case-based data. Additionally, our in-depth interviews were restricted to a few key stakeholders in hospital settings and insurance representatives and hence, could suffer from selection and information biases. However, our nature of enquiry was exploratory in nature and not intended to generalize findings. We believe a more comprehensive understanding of injury in India can be achieved by understanding policy and programme implementation bottlenecks. For multi-source data, further understanding of the processes in police and insurance data source systems is needed. Despite the above limitations, our review, probably represents first such review of injury surveillance systems in India. Hence, our mapping of data sources and identification of gaps and opportunities may help in developing plans and further studies.

## Conclusions

Based on the review, we identified that India has multiple data sources at various levels in varying capacities that could potentially contribute to an effective injury surveillance system in India. Secondly, we concluded that the existing reporting systems for injury mortality and morbidity lack reliability and validity due to non- standardized data collection, classification and reporting methods. Thirdly we identified that risk factor data is non-existent in India in routinely collected data, although may be available in specific research studies.

Robust and tested tools exist that may be adopted to the context for comparable data [9]. To improve injury prevention and control efforts across the country, we recommend creating a standard operating procedure for data collection and reporting and supported by training. We suggest identification of bottlenecks for implementation for injury reporting systems through consultations with stakeholders across meso and macro level.

Finally, we suggest that accountability for monitoring and evaluating injury surveillance system may require designating a dedicated authority for instance an Injury Surveillance Nodal Officer, within The Ministry of Health and Welfare, with co-ordination roles across multiple other Ministries such as Road and Transport, and The Planning Commission of India. Further buy in and commitment from multiple agencies with accountability measures need to be built for implementation of a comprehensive injury surveillance system.

## Supplementary Information

**Additional file 1: Table S1.** Grey literature data sources and key findings for injury morbidity and mortality in India. **Table S2.** National and international data sources of injury morbidity and mortality in India rated according to attributes of injury surveillance. **Supplementary Table 3.** Status of WHO core and optional variables collected at data sources. Adapted WHO injury surveillance evaluation tool.

## Data Availability

The datasets during and/or analysed during the current study available from the corresponding author on reasonable request.

## Abbreviations

ASHA: Accredited Social Health Activist
GBD: The Global Burden of Diseases Study
ICD: International Classification of Diseases
PGIMER: Postgraduate Institute of Medical Education and Research Chandigarh
WHO: The World Health Organization
