# Supplementary material for: Potential for establishing an injury surveillance system in India: a review of data sources and reporting systems
Source: BMC Public Health. 2020 Dec 14;20:1909. doi: 10.1186/s12889-020-09992-9 (PMC7734854; doi:10.1186/s12889-020-09992-9)
Supplement: Supplementary file 1 — Additional file 1: Table S1. Grey literature data sources and key findings for injury morbidity and mortality in India. Table S2. National and international data sources of injury morbidity and mortality in India rated according to attributes of injury surveillance. Supplementary Table 3. Status of WHO core and optional variables collected at data sources. Adapted WHO injury surveillance evaluation tool. [file 12889_2020_9992_MOESM1_ESM.docx]

**Supplementary Table 1: Grey literature data sources and key findings for injury morbidity and mortality in India**

| **Source** | **Year** | **Source of data** | **Population** | **Definitions** | **Main findings** | **Website and date accessed** | **Strengths/Limitations** |
| --- | --- | --- | --- | --- | --- | --- | --- |
| **Road/Traffic Injuries** |  |  |  |  |  |  |  |
| **National Crime Research Bureau (NRCB)**  **Ministry of Home Affairs** | 2018 | Police registration of injury cases | All India | Traffic accidents are comprised of ‘road accidents’ and  ‘Railway crossing accidents’.  (Railway accidents are reported separately). | In 2018 there were 4,74,638 traffic accidents  (4,45,514 road accidents, 27,643  railway accidents and 1,481 railway crossing  accidents) these accidents  caused 1,52,780, 24,545 and 1,507 deaths  respectively and resulted in injuries  to 4,49,981 persons.  Analysis of traffic accidents reveal that maximum number of ‘Traffic Accidents’ have taken place during 18:00 hrs – 21:00 hrs and 15:00 hrs – 18:00 hrs (18.7% ) , 88,792 out of 4,74,638) and (17.0%, 80,746 out of 4,74,638) of total traffic accidents respectively  Two wheelers have accounted for maximum fatal road accidents (54,610 deaths, 35.7% of total road accident deaths), followed by trucks/lorries 15.9% (24,260 deaths) cars (21,290 deaths) (13.9%) and buses (10,514 deaths) (6.9%).  Speeding accounted for 59.3% of total accidents (2,64,158 out of 4,45,554 cases) which caused 84,346 deaths and injuries to 2,74,997 persons. | <http://www.ncrb.gov.in/>  Accessed: 12/03/2020 | (+) National level, ongoing, good source of trend data.  (+) disaggregates sex, includes type of vehicle involved, type of road, time of occurrence, cause of accident etc  (-)The cases reported are the ones registered under Indian Penal Code. A much higher number of cases are reported in police daily diaries/First Incident Report (FIR) which are not reported beyond the station. Leading to gross under- reporting. |
| **Ministry of Road, Transport and Highways**  **(MORTH)** | 2017 | Data from state roads and highways, cases identified through police reports using 19 items format based on Asian Pacific Road Accident data/ Indian Road Accident data. | All India | No definition provided. | In 2017 464,910 road accidents were recorded. There were 147,913 road accident deaths (86.4% male; 13.6% female). This was a 2,814 increase in deaths from 2016.  In 2017 26.92% of deaths were on State Highways, 35.95% on National Highways and 37.13% on ‘other’.  The largest number of deaths from road accidents were among 25-34 year olds (39,548.93), followed by 18-25 year olds (34,243.70). | <http://morth-roadsafety.nic.in/report.asp>  Accessed: 7 June 2019 | (+) Transport Research Wing can be useful resource for surveillance of road traffic injuries. It also compiles data on the registered motor vehicles from States/UTs.  (+) Online data base is in place.  (-) The data source relies on the NCRB data above and hence has same limitations. |
| **Office of the Registrar General & Census Commissioner**  **Ministry of Home Affairs** | 2010 - 2013 | Cause of death statistics-  Nationally representative household survey (rural & urban now combined) | All India | As per ICD-10 codes:  Motor vehicle accidents: V01-V89 | Between 2010-2013 there were 5373 deaths from motor vehicle accidents recorded (861 female; 4512 male). They made up 27.5% of the total burden of 19,563 injury deaths. And 2.9% of the 182, 827 total deaths.  The highest proportion of deaths from motor vehicle accidents were among 15-29 year olds (13.7%) followed by 30-44 year olds (9.0%). | <http://www.censusindia.gov.in/2011-Common/CensusInfo.html>  Accessed: 21 June 2019 | (+) Community based data. Good validity.  (-) Only cause of death ascertainment. Sample is representative only at National level. Good source for mortality burden but insufficient to guide development of interventions  (-) Every 10 years |
| **National Injury Surveillance Trauma Registry and Capacity Building Centre** | 2016  Pilot -  (1st March to 31March 2016). | Hospital based data from Dr. RML Hospital. | New Delhi – 12 hospitals sites proposed | Guided by WHO injury and trauma data collection guidelines. | Data from report (1 month only), 115 records of road traffic injuries. The highest proportion of patients (overall) are male (78%) female (22%) and transgender (0%). | [http://www.nisc.go](http://www.nisc.gov.in/Mission.aspx) [v.in/Mission.aspx](http://www.nisc.gov.in/Mission.aspx)  Accessed: 7 June 2019 | (+)The resource provides an opportunity to collect data in a standardized, online- timely manner.  (-) There is room for simplification of the form and putting in place some internal validity check to ensure data quality. |
| **Medically Certified Cause of Death (MCCD)**  **(Registrar General of India)** | 2015 | Medically certified cause of death (MCCD) records. | All India | As per ICD-10 codes:  Railway accidents: V05,V15,V80.6,V81,V82.2, V87.6 & V88.6  Motor vehicle accidents: V02-V04, V09.2-09.3,  V19.6, V19.9, V20-V28, V29.4-V29.6, V29.9, V30- V38, V39, 39.9, V40- V48, V49.4- V49.6,49.9, V50-V58, V59.4-V59  Other road vehicle: V01, V06, V09.9, V10-V11, V16- V18, V19.8, V29.8, V39.8,  V49.8, V59.8,  V69.8, V79.8,  V80.0-V80.2,  V80.7-V80.9,  V82.3-V82.7, V82.9 & V89.1.  Water transport: V90-  94. Air & Space transport: V95- V97. All other transport: V09.0-V09.1, V19.0-V19.3, V29.3, V39.0- V39.3, V49.0- V49.3, V59.0- V59.3, V69.0- V6, V79.3, V82.0, V82.8, V83-V86, V88.0-V88.5, V88.7-V88.9. | 10272 deaths were from motor vehicle traffic accidents. A smaller proportion were in women; n= 2115, 21% female (n= 8157, 79% male).  Other road vehicle accidents account for 2428 deaths (486 female and 1942 male). | <http://www.censusindia.gov.in/2011-Documents/mccd_Report1/MCCD_Report-2015.pdf>  Accessed: 7 June 2019 | (-) missing data on mechanism of injury.  (-) 10-15% of all deaths are unclassifiable  (-) 10% deaths reported have inaccurate reporting of deaths in various age and sex categories.  (-) The completeness of data from the states varies, covering between less than 1% deaths in Uttar Pradesh to 100 % in Goa in 2015.  (-) Does not record deaths at home. |
| **Ministry of Statistics**  **(NSSO National Sample Survey Organisation)** | 2014 | National Sample Survey | India | Number of self-reported hospitalisations resulting from injury. Injury which was not deliberate but accidental leading to lacerations, fractures, crushing injuries, injuries to internal organs or multiple body parts | In 2014 there were 350 hospitalisations per 100,000 persons across India from accidental injury, road traffic accident & falls.  Of all injuries 48% used public health facility and 52% private. | <http://www.mospi.gov.in/national-sample-survey-office-nsso-0>  Accessed: 25 July 2019 | (+) Provides community based data on hospitalization, every 10th year. Provides information on cost and utilization of public vs private health a facilities.  The results suggest that public private partnership is essential for responsive trauma care.  (-) Classification and coding not comparable with other data sources. Could be prone to recall bias.  (-) Only every ten years |
| **India Open Data Platform / Open Government Data (OGD) Platform** | 2017 | Data from the Transport Research Wing (TRW) of Ministry of Road Transport & Highways from the Police Headquarters of the various states | All India | No definition provided (as per Ministry of Road Transport & Highways) | Same as Ministry of Road Transport and Highways data, refer to above. | <https://data.gov.in/resources/stateut-wise-total-number-road-accidents-persons-killed-and-injured-other-roads-during>  Accessed: 18 June 2016 | (+) The resource provides an open data platform for compilation of data from various resources.  (-) Relies on police data. Definitions and methods are unclear to comment on system. |
| **Government of India Ministry of Statistics and program Implementation**  **(MOSPI)** | 2015 | SDG Baseline Report  2019  The data source is National Crime Record Bureau:  ‘Accidental Deaths & Suicides in India 2015‟ | All India | Death rate due to road traffic accidents (Indicator 3.6.1) | In 2015 the death rate due to road traffic accidents was reported as 11.81/ 100,000. | [http://www.mospi.gov.in/#](http://www.mospi.gov.in/)  Accessed: 21 June 2019 | (-) Data from NRCB, same limitations apply. |
| **Global Status Report on Road Safety 2018** | 2016 | Data from Ministry of Road Transport and Highways, Road Accidents. | Urban –rural population based data. | Motor vehicle accidents: V01-V89 | In 2016 total reported road traffic fatalities were 150,785 . The greatest proportion of deaths were in males (85% male, 15% female).  Four percent of road traffic deaths involved alcohol.  The number of WHO estimated road traffic fatalities in 2016 was 299,091. | <https://www.who.int/violence_injury_prevention/road_safety_status/2018/en/>  Accessed: 18 June 2019 | (+) Regular timely reports  (+) WHO estimates were presented as well as reported figures to account for under reporting and give idea of magnitude.  (+) A good source of data on risk factors  (~) Extrapolated data from MORTH |
| **Global Burden of Disease**  **(GBD)**  **(Institute of Health Metrics and Evaluation, IHME)** | 2017 | Extrapolates data using MCCD and SCD, and SRS data | Urban –rural population based data. | Motor vehicle accidents: V01-V89 | An estimated total number 1,498,496.86 transport injuries were sustained in 2017.  Transport injury caused a total of 244,710.95 deaths (187,791.56 male; 56,919.38 female).  In 2017 road injury caused 218,876 deaths. Three quarters of this burden was in males (n= 167,829, 76%. Females accounted for 23%, (n= 51,046). | [http://ghdx.healthd](http://ghdx.healthdata.org/gbd-results-tool) [ata.org/gbd-](http://ghdx.healthdata.org/gbd-results-tool) [results-tool](http://ghdx.healthdata.org/gbd-results-tool)  Accessed: 21 June 2019 | (~) extrapolated data |
| **Young Lives Study** | 2009-10 | Longitudinal qualitative study | Rural urban sites in Andhra Pradesh | Injury reported by children/carers during interviews with injury closed question on limited mechanisms | 18.5% (336/1820) of children aged 7-9 years had a serious non-fatal injury. Injury incidence was 21.5% among males; 15.0% among females. 14.9% (n=50) of injuries among children aged 7-9 years were from RTIs | [http://www.youngli](http://www.younglives.org.uk/) [ves.org.uk/](http://www.younglives.org.uk/)  Accessed: 15 December 2016  (No updated data on injury available when accessed again on:  21/06/2019) | (+)A prospective community based longitudinal study  (-) However recall period is 2-3 years.  (-) Methods to capture injury data not robust |
| **Fall Injury** |  |  |  |  |  |  |  |
| **National Crime Research Bureau**  **(Ministry of Home Affairs, India)** | 2015 | Police registration of injury cases | All India | ‘Falls’ refer to falls from height, falls from vehicles, fall into manhole, fall into pit, fall into bore well and others | In 2015, there were 17,433 cases of falls reported in India. Of these cases, 969 (5%) resulted in non-fatal injury 16,759 (95%) resulted in death.  The most common mechanism of fall was ‘fall from a height’, accounting for 54% (n=9422) of all falls, followed by ‘Fall from Vehicles (automobile like bus, trucks etc.)’, accounting for 9% (n=1625) of all falls. A significant number of falls were classified to occur in ‘other’ circumstances (32%, n=5556).  A larger proportion of fatal falls occurred among men (n= 13968, 83%) when compared to women (n= 2790, 17%).  The largest number of fall deaths was reported for people aged between 30 and 45 years (n= 5151, 31%), followed by people aged 18 to 30 years (n= 4115, 25%).  The largest number of fall deaths were recorded for the state of Maharashtra (n= 3314, 20%), followed by Gujarat (n= 2309, 14%) and Madhya Pradesh (n= 1487, 9%).  Fatal falls have increased by 8.8% from the 2014 NCRB data.  Falls accounted for 5% of accidental injury deaths and fall-related injuries accounted for 0.2% of all injuries recorded in 2015. | <http://www.ncrb.gov.in/>  Accessed: 7 June 2019 | (-) In addition to reporting bias in general, falls would be highly under- reported as same level falls not included. |
| **Medically Certified Cause of Death**  **(Registrar General of India)** | 2015 | Medically certified cause of death records | All India | As per ICD-10 codes:  Accidental falls: W00-W19 | In 2015, there were 2201 deaths from accidental falls recorded in medical certificates, with a larger number occurring among men (n=1629, 74%) when compared to women (n=-572, 26%). The largest number of fall deaths was documented for Delhi (n=494, 22%).  The largest number of fall deaths occurred among people aged 25 to 34 years (n=393, 18%), followed by those aged 45 to 54 years (n=347, 16%). | <http://www.censusindia.gov.in/2011-Documents/mccd_Report1/MCCD_Report-2015.pdf>  Accessed: 7 June 2019 | (+) Routinely collected data that with quality improvement can provide data on moderate to severe injuries requiring hospitalisation in urban areas. Standardised/ comparable codes to other global data  (-) 22% of total registered deaths in 2015 were medically certified -grossly under-reported. |
| **Ministry of Labour and Employment** | 2016 | Database of mine safety – from inquiries and inspections. | Miners | ‘fall of person’ | In 2016 9.7% of fatal accidents in coal mines were due to ‘fall of person’. In 2016 34% of serious accidents in coal mines were due to ‘fall of person’. There were 5 fatal falls in coal mines in 2016 (increased 25%, 4 in 2015).  There were 87 serious accidents involving a ‘fall of person’ in coal mines (decreased 17%, 105 in 2015).  21.4% of fatal accidents in non- coal mines in 2016 were due to ‘fall of person’. 37.4% of serious accidents in non-coal mines were due to ‘fall of person’. There were 8 fatal falls in 2016 in non-coal mines (decreased by 11%, 9 in 2015). There were 14 serious ‘fall of persons’ accidents in non-coal mines in 2016 (increased 280%, from 5 in 2015). | <https://labour.gov.in/sites/default/files/ANNUAL_REPORT_2017-18-ENGLISH.pdf>  Accessed: 23 July 2018 | (-) only reporting falls in coal mines  (+) reported annually – can see trends |
| **Ministry of Statistics**  **(NSSO)** | 2014 | National Sample Survey | All India | Number of self- reported hospitalisations resulting from injury | 350 hospitalisations per 100,000 persons across India from accidental injury, road traffic accident & falls combined. (Separate data for falls not available). | <http://www.mospi.gov.in/national-sample-survey-office-nsso-0>  Accessed: 25 July 2019 | (+) Provides community based data on hospitalization, every 10th year.  (-) data only available every10 years  (+) Provides information on cost and utilization of public vs private health a facilities.  (-) Classification and coding not comparable with other data sources. Could be prone to recall bias. |
| **Open Government Data (OGD) Platform** | 2003-  2014 | Police registration of injury cases (same data as reported by NCRB) | All India | No definition provided | 15,399 fall-related deaths occurred in 2014. | <https://data.gov.in/catalog/stateut-wise-distribution-accidental-deaths-un-natural-causes>  Accessed: 19 July 2019 | (+) Primary resource is NCRB, however has potential under [National](https://data.gov.in/sites/default/files/NDSAP.pdf) [Data Sharing and](https://data.gov.in/sites/default/files/NDSAP.pdf) [Accessibility Policy](https://data.gov.in/sites/default/files/NDSAP.pdf) [(NDSAP)](https://data.gov.in/sites/default/files/NDSAP.pdf) to compile data from serval sources.  (+)State wide data from 2001- 2012 is available. |
| **National Injury Surveillance Trauma Registry and Capacity Building Centre** | 2016-  pilot  (1st March to 31March 2016). | Hospital based data from Dr. RML Hospital. | New Delhi 12 hospitals sites proposed | Guided by WHO injury and trauma data collection guidelines. | Data from 1 month only, 24 falls out of 167 total reports.  22% of patients overall are female; 78% are male and 0% are transgender (not fall specific sex disaggregation). | [http://www.nisc.go](http://www.nisc.gov.in/Mission.aspx) [v.in/Mission.aspx](http://www.nisc.gov.in/Mission.aspx)  Accessed: 7 June 2019 | (+)The resource provides an opportunity to collect data in a standardized, online- timely manner.  (-) There is room for simplification of the form and putting in place some internal validity check to ensure data quality. |
| **Global Burden of Diseases Study**  **(Institute of Health Metrics and Evaluation)** | 2017 | Estimates generated based on secondary data sources | All India | Based on ICD 10 coding. Deaths and DALY’s | In 2017, 221,297 fall deaths were reported for India, with slightly more occurring among women (n=117,352, 53%) than men (n=103,945, 47 %). A total of 8,020,326 DALYs were lost due to falls.  The greatest number of fall deaths were reported among people aged 70 years and above (n= 123,590, 56%), followed by people aged 50-69 years (n=55,677, 25%). | [http://ghdx.healt](http://ghdx.healthdata.org/gbd-results-tool) [hdata.org/gbd-](http://ghdx.healthdata.org/gbd-results-tool) [results-tool](http://ghdx.healthdata.org/gbd-results-tool)  Accessed: 20 June 2019 | (~) Extrapolated data based on secondary data sources with their own limitations. |
| **Young Lives Study** | 2009-2010 | Longitudinal qualitative study | Andhra Pradesh | Injury reported by children/carers during interviews | 18.5% (336/1820) of children aged 7-9 years had a serious non-fatal injury. Injury incidence was 21.5% among males; 15.0% among females. 186 (55.4%) children reported injury from a fall. Over half of injuries in this age group were a result of falls. | [http://www.youn](http://www.younglives.org.uk/) [glives.org.uk/](http://www.younglives.org.uk/)  Accessed: 15  Dec 2016  (No updated data on injury available when accessed again on:  21/06/2019) | (+) A prospective community based longitudinal study  (-) However recall period is 2-3 years.  (-) Methods to capture injury data not robust |
| **Fires/ Burn Injury** |  |  |  |  |  |  |  |
| **National Crime Research Bureau**  **(Ministry of Home Affairs)** | 2015 | Police registration of injury cases | All India | ‘Accidental fire’ refers to fires from electrical short circuit, riot/agitation, fireworks, cooking gas/cylinder/stove burst and other causes | In 2015, 18,450 cases of accidental fires were reported, this is a 9.5% decrease from 2014.  These fires resulted in 1,193 injuries and 17,700 deaths, almost two thirds (62%) of this burden was on females (n=10,925, n=6775 males and n= 0 transgender).  42% of the fire deaths (n=7445) were in the residential dwelling/building.  22% of fire accidents were recorded in Maharashtra (n= 4,087 out of 18,450).  A total of 58 cases of accidental fire in trains were also reported during 2015 which caused 59 deaths in the country during 2015.  Accidental fire accounted for 4.2 % of accidental deaths in 2015. | <http://www.ncrb.gov.in/>  Accessed: 7 June 2019 | (+) National level, ongoing, good source of trend data.  (+) Disaggregates sex to include transgender  (+) State by state analysis of number of fires and location of fires contributes meaningful data to prevention efforts.  (-) The cases reported are the ones registered under Indian Penal Code. A much higher number of cases are reported in police daily diaries/Fist Incident Report (FIR) which are not reported beyond the station. Leading to gross under- reporting. |
| **Medically Certified Cause of Death**  **(Registrar General of India)** | 2015 | Medically certified cause of death records | Urban hospitals | As per ICD-10 codes:  Exposure to smoke fire and flames: X00-X09  Burns and corrosion: T20- T32 | Exposure to smoke, fire and flames caused a total of 4,406 deaths. There is a slightly higher proportion of women who died (54%,(n= 2396 ) than men (n= 2010 )  Burns & corrosions caused 13,238 deaths in 2015. 41% were in males and 59% females (n= 5453; n= 7785 respectively).  Burns and Corrosions contributed 17.9 % of the total deaths under the ‘Injury, Poisoning & Certain Other Consequences of External Causes’ group. 1.1% of the total medically certified deaths. | <http://www.censusindia.gov.in/2011-Documents/mccd_Report1/MCCD_Report-2015.pdf>  Accessed: 7 June 2019 | (-) 10-15% of all deaths are unclassifiable  (-) 10% deaths reported have inaccurate reporting of deaths in various age and sex categories.  (-) The completeness of data from the states varies, covering between less than 1% deaths in Uttar Pradesh to 100 % in Goa in 2015.  (-) Does not record deaths that occurred at home. |
| **Ministry of Labour and Employment** | 2016 | Database of mine safety – from inquiries and inspections | mines | ‘gas, fire, dust, etc.’ | In 2016 4 fatal reports under this category and 0 serious accidents in coal mines. No disaggregation on how many deaths pertained to fire. | <https://labour.gov.in/sites/default/files/ANNUAL_REPORT_2017-18-ENGLISH.pdf>  Accessed: 25 July 2019 | (-) fire category combined with dust and gas.  (-) only in mines  (+) annual report.  (+) mandatory reporting of serious incidents  (~) Electrical and Chemical burns in specific related to occupational injuries are relevant. The Ministry might have a capacity to look into risk factors specific to occupational injuries |
| **Ministry of Statistics**  **(NSSO)** | 2014 | National Sample Survey | All India | All self-reported hospitalisation for burns, or corrosions due to fire, steam/vapour, hot liquids, acids or chemicals leading to boils, abrasions and lacerations | In 2014 the rate of hospitalisations for burns and corrosion injuries was 15 hospitalisations per 100,000 persons across India. | <http://www.mospi.gov.in/national-sample-survey-office-nsso-0>  Accessed: 25 July 2019 | (~) The numbers were small to compare costs associated data, however literature suggests that costs associated with burns are high, with need for prolonged rehabilitation. |
| **National Injury Surveillance Trauma Registry and Capacity Building Centre** | 2016- pilot  (1st March to 31March 2016). | Hospital based data from Dr. RML Hospital. | New Delhi 12 hospitals sites proposed | Guided by WHO injury and trauma data collection guidelines. | 5 burns out of a total 167 reports  (1 month only).  22% of patients overall are female; 78% are male and 0% are transgender. | [http://www.nisc.go](http://www.nisc.gov.in/Mission.aspx) [v.in/Mission.aspx](http://www.nisc.gov.in/Mission.aspx)  Accessed: 7 June 2019 | (+)The resource provides an opportunity to collect data in a standardized, online- timely manner.  (-) There is room for simplification of the form and putting in place some internal validity check to ensure data quality. |
| **Open Government Data Platform (OGD)** | 2003-2014 | Police registration of injury cases (same data as reported by NCRB) | All India, state specific data from 2001-  2012 available | No definition provided | There were 19,513 fire related deaths in 2014. | [https://data.gov.in/](https://data.gov.in/catalog/stateut-wise-distribution-accidental-deaths-un-natural-causes) [catalog/stateut-](https://data.gov.in/catalog/stateut-wise-distribution-accidental-deaths-un-natural-causes) [wise-distribution-](https://data.gov.in/catalog/stateut-wise-distribution-accidental-deaths-un-natural-causes) [accidental-deaths-](https://data.gov.in/catalog/stateut-wise-distribution-accidental-deaths-un-natural-causes) [un-natural-causes](https://data.gov.in/catalog/stateut-wise-distribution-accidental-deaths-un-natural-causes)  Accessed: 21/10/19 | (-) Relies on NCRB data and prone to limitations of the primary data source |
| **Global Burden of Diseases Study**  **(Institute of Health Metrics and Evaluation)** | 2017 | Estimates generated based on secondary data sources | All India | Deaths or DALYs from fire, heat or hot substances | An estimated 27,026.99 deaths from fire/heat/hot substances (7,161.17 males; 19,865.82 females).  Fire/heat/hot substances caused an estimated 1,989,247.28 DALYs. | [http://ghdx.healthd](http://ghdx.healthdata.org/gbd-results-tool) [ata.org/gbd-](http://ghdx.healthdata.org/gbd-results-tool) [results-tool](http://ghdx.healthdata.org/gbd-results-tool)  Accessed: 21 June 2019 | (~) Extrapolated data based on secondary data sources with their own limitations. |
| **Drowning Injury** |  |  |  |  |  |  |  |
| **National Crime Research Bureau**  **(Ministry of Home Affairs)** | 2015 | Police registration of injury cases | All India | Drowning deaths and injuries refer to those resulting from boat capsize, accidental falls into water body and other causes | 29,822 drowning deaths (23,163 male; 6654 Female and 5 transgender) accounting for 8.9% of all accidental deaths.  29,232 cases of drowning reported with 481 drowning-related injuries. | <http://www.ncrb.gov.in/>  Accessed: 7 June 2019 | (+) The definition includes most recent drowning inclusion with respect to transport- based on recommendation by Passmore et al.  (+) disaggregates sex to include transgender  (-) In addition to above highlighted limitations, the population based data reports high burden in children under 5 years, post disaster’s such as flooding and so are likely to be grossly under-reported with little medico – legal implications. |
| **Medically Certified Cause of Death**  **(Registrar General of India)** | 2015 | Medically certified cause of death records | All India | As per ICD-10 codes: Accidental drowning and submersion: W65-W74 | Accidental drowning and submersion caused a total of 1476 deaths.  The largest proportion (75%) of drowning deaths were amongst males (n= 1103) with only 25% amongst females (n=373). | <http://www.censusindia.gov.in/2011-Documents/mccd_Report1/MCCD_Report-2015.pdf>  Accessed: 7 June 2019 | (-) Drowning deaths are almost always immediate, in addition to limitations of hospital based data in general – drowning cases are unlikely to be presented at hospitals. |
| **Ministry of Environment, Forest and Climate Change** |  |  |  |  |  | [http://envfor.nic.i](http://envfor.nic.in/) [n](http://envfor.nic.in/)  Accessed: 21 June 2019 | (~) Whilst no data is being collected on drowning as per the current report, there is suggestion for impact of climate change. |
| **Ministry of Statistics**  **(NSSO)** | 2014 | National Sample Survey | Number of hospitalisations resulting from injury | Accidental drowning and submersion | The rate of hospitalisations for drowning or submersion was 3 hospitalisations / 100,000 persons across all India | [http://www.india](http://www.indiaenvironmentportal.org.in/files/file/health%20in%20India.pdf) [environmentport](http://www.indiaenvironmentportal.org.in/files/file/health%20in%20India.pdf) [al.org.in/files/file/](http://www.indiaenvironmentportal.org.in/files/file/health%20in%20India.pdf) [health%20in%2](http://www.indiaenvironmentportal.org.in/files/file/health%20in%20India.pdf) [0India.pdf](http://www.indiaenvironmentportal.org.in/files/file/health%20in%20India.pdf)  Accessed: 25 July 2019 | (~) Little is known on drowning related morbidity. Though data source has its limitations (as highlighted in the above table) it provides some information on hospitalisation. |
| **Open Government Data Platform** | 2014 | Police registration of injury cases  (Uses data from Accidental Deaths and Suicides in India 2014, National Crime Records Bureau (NCRB) | All India | Drowning deaths and injuries refer to those resulting from boat capsize, accidental falls into water body and other causes | In 2014 there were 29,903 drowning related deaths. | [https://data.gov.i](https://data.gov.in/catalog/stateut-wise-distribution-accidental-deaths-un-natural-causes) [n/catalog/stateut](https://data.gov.in/catalog/stateut-wise-distribution-accidental-deaths-un-natural-causes)  [-wise-](https://data.gov.in/catalog/stateut-wise-distribution-accidental-deaths-un-natural-causes) [distribution-](https://data.gov.in/catalog/stateut-wise-distribution-accidental-deaths-un-natural-causes) [accidental-](https://data.gov.in/catalog/stateut-wise-distribution-accidental-deaths-un-natural-causes) [deaths-un-](https://data.gov.in/catalog/stateut-wise-distribution-accidental-deaths-un-natural-causes) [natural-causes](https://data.gov.in/catalog/stateut-wise-distribution-accidental-deaths-un-natural-causes)  Accessed: 21 June 2019 | (-) Limitations are same as the primary source of data.  (+)State wide data from 2001-2012 available  (+) State-wide distribution of accidental deaths by un- natural causes during 2014 |
| **Global Burden of Diseases Study**  **(Institute of Health Metrics and Evaluation)** | 2017 | Estimates generated based on secondary data sources | All India | Deaths or DALYs from drowning or submersion | For 2017 in all of India there were an estimated total of 60,198 deaths from drowning.  The largest proportion of drowning deaths were in males (64%) n= 38,594, and females (36%) n=21,603.  Drowning caused an estimated 3,372,855 DALYs in 2017. | [http://ghdx.healt](http://ghdx.healthdata.org/gbd-results-tool) [hdata.org/gbd-results-tool](http://ghdx.healthdata.org/gbd-results-tool)  Accessed: 21 June 2019 | (~) Extrapolated data based on secondary data sources with their own limitations. |
| **WHO Global Report on Drowning** |  |  |  |  | No national level data available | [http://www.who.i](http://www.who.int/violence_injury_prevention/global_report_drowning/en/) [nt/violence_injur](http://www.who.int/violence_injury_prevention/global_report_drowning/en/) [y_prevention/glo](http://www.who.int/violence_injury_prevention/global_report_drowning/en/) [bal_report_drow](http://www.who.int/violence_injury_prevention/global_report_drowning/en/) [ning/en/](http://www.who.int/violence_injury_prevention/global_report_drowning/en/)  Accessed: 21 June 2019 |  |
| **Forces of nature Injury** |  |  |  |  |  |  |  |
| **National Crime Research Bureau**  **(Ministry of Home Affairs)** | 2015 | Police registration of injury cases | All India | Causes attributable to forces of nature: avalanche, exposure to cold, landslide, torrential rain and heat/sun stroke, cyclone, tornado, tsunami, starvation due to natural calamity, earthquake, epidemic, flood, lightning, forest fire, torrential rain, other natural cause | 10,510 deaths recorded in 2015. | <http://www.ncrb.gov.in/>  Accessed on:  07/06/2019 | (+) National level data system in place and good source of trend data.  (-) Prone to under- reporting and data not comparable to other data sources. |
| **Government of India Ministry of Statistics and program Implementation** | 2015-  2016 | SDG baseline report 2019  Data Source for this indicator is Ministry of Home Affairs | All India | SDG indicator 1.5.1  Number of deaths, missing persons and directly affected persons attributed to disasters per 100,000 population | Not Available | [http://www.mospi.gov.in/#](http://www.mospi.gov.in/)  Accessed: 21 June 2019 | (-) No data available |
| **Global Burden of Diseases Study**  **(Institute of Health Metrics and Evaluation)** | 2017 | Estimates generated based on secondary data sources | All India | Deaths or DALYs from exposure to forces of nature | An estimated total of 1,979.02  deaths caused by forces of nature (1,249 males; 730 females)  An estimated 98,290 DALYs caused by exposure to forces of nature. | [http://ghdx.healthdata](http://ghdx.healthdata.org/gbd-results-tool)  [.org/gbd-results-tool](http://ghdx.healthdata.org/gbd-results-tool)  Accessed: 21 June 2019 | (~) Extrapolated data based on secondary data sources with their own limitations. |
| **Occupational causes** |  |  |  |  |  |  |  |
| **National Crime Records Bureau** | 2015 | Police registration of injury cases | All India | Occupational causes are referred to as: ‘factory/machine accidents’ and ‘mine/quarry disasters’ | In 2015 1268 cases of factory/machine accidents were reported. Leading to 594 injured people. The largest proportion was in males 93%, n= 551. Females accounted for 6.7%, n= 40 and 0.5% n=3 in transgender people.  In 2015, 695 people died in factory accidents (88%, n=613 males; 12%, n=82 females and 0%, n=0 transgender).  In 2015, 119 cases of mine/quarry disasters were reported. These injured 8 people (6 male, 2 female, 0 transgender) and killing 0. | <http://www.ncrb.gov.in/>  Accessed: 7 June 2019 | (-) Limitations as above. It is likely that injuries from organised sector are well reported whilst unorganised sector is grossly under- reported.  (+) disaggregates sex to include transgender |
| **Ministry of Labour and Employment** | 2013 | Labour Bureau Shimla, Ministry of Labour and Employment  Statistical Year Book India 2017 | All India  The coverage remains limited with partial reporting from States | Figures relate to compensation of injuries under Employee's Compensation Act. | 2756 employee deaths compensated under the Compensation Act.  3078 injuries resulting in permanent disability received compensation and 1658 temporary injuries received compensation. | <http://mospi.nic.in/statistical-year-book-india/2017>  Accessed:21 June 2019 | (-) Lacks standardization and timely reporting  (-)Yearly figures may not be strictly comparable due to difference in area covered |
| **Open Government Data Platform** | 2013 | Data from Ministry of Labour and Employment | All India (but not all states reported - data from 16/30 states ) | The number of persons receiving injuries as a result of an accident. | 1,951 non- fatal factory injuries reported and 494 fatal factory injuries | [https://data.gov.](https://data.gov.in/catalog/industrial-injuries-factories) [in/cat](https://data.gov.in/catalog/industrial-injuries-factories) [alog/industrial-](https://data.gov.in/catalog/industrial-injuries-factories) [injuries-](https://data.gov.in/catalog/industrial-injuries-factories) [factorie](https://data.gov.in/catalog/industrial-injuries-factories)s  Accessed: 21 June 2019 | (-) Limitations are same as the primary source of data.  (-)The data presented relates only to factories submitting returns.  (+)Trend data is available for 2007- 2013 |
| **Government of India Ministry of Statistics and program Implementation** | 2013 | SDG baseline report 2019  The data source for this indicator is Ministry of Labour and Employment | All India | The indicator (8.8.3) is defined as number injuries resulting in fatal and non-fatal accidents in factories. | Number of accidents in factories 2,445 (fatal 494 non-fatal 1,951) | [http://www.mospi.gov.in/#](http://www.mospi.gov.in/)  Accessed: 21 June 2019 | (-) Secondary data from Ministry of Labour and employment |
| **Other Injuries** |  |  |  |  |  |  |  |
| **Integrated Disease Surveillance Program**  **Ministry of Health and Family Welfare** | 2016 | Hospital based | All India | Dog bite Snake Bite | Reports on weekly basis, latest data available from May 2019. No dog or snake bites recorded. | <http://www.idsp.nic.in/>  Accessed:21 June 2019 | (-) No injury data recorded except dog/snake bite.  (+) This is an integrated well developed surveillance system that has the potential to be expanded for hospitalised injury data  (+) 97% Districts are reporting weekly disease surveillance data under IDSP |
| **Ministry of Statistics**  **(NSSO)** | 2014 | National Sample Survey | Number of hospitalisations resulting from injury | Poisoning - Internal ingestion of excessive inappropriate levels of medicines, any levels of pesticides, insecticides, rat poisons or other chemicals, applications on skin.  Intentional self-harm – suicide, attempted suicide or even deliberate self injury inflicted on oneself for whatever reason.  Assault - Harm inflicted deliberately by another human being.  Contact with animals/plants- Snake-bites, scorpion stings, any other insect bite, any other animal bit – dogs, wild animals  Accidental poisoning or contact with plants – excludes that done with suicidal intent | Poisoning rate 17/100,000.  Rate of Intentional self- harm 5/100,000  Rate of injury from assault 7/100,000  Rate of injury from contact with venomous/harm-causing animals and plants 20/100,000.  Total rate (of all injuries combined including drowning etc.) 416/100,000 | [http://www.india](http://www.indiaenvironmentportal.org.in/files/file/health%20in%20India.pdf) [environmentport](http://www.indiaenvironmentportal.org.in/files/file/health%20in%20India.pdf) [al.org.in/files/file/](http://www.indiaenvironmentportal.org.in/files/file/health%20in%20India.pdf) [health%20in%2](http://www.indiaenvironmentportal.org.in/files/file/health%20in%20India.pdf) [0India.pdf](http://www.indiaenvironmentportal.org.in/files/file/health%20in%20India.pdf)  Accessed: 25 July 2019 | (~) Though data source has its limitations – only hospital presentations. It provides some information on hospitalization. |
| **National Injury Surveillance Trauma Registry and Capacity Building Centre** | 2016- pilot  (1st March to 31March 2016). | Hospital based data from Dr RML Hospital. | New Delhi 12 hospitals sites proposed | Guided by WHO injury and trauma data collection guidelines. | Data from report (1 month only) show 22% of patients overall are female; 78% are male and 0% are transgender.  12 assaults out of 167 total injury reports. | <http://www.nisc.go> [v.in/Mission.aspx](http://www.nisc.gov.in/Mission.aspx)  Accessed: 7 June 2019 | (+)The resource provides an opportunity to collect data in a standardized, online- timely manner.  (-) Whilst online data form could not be accessed-from the available PDF there is room for simplification of the form and putting in place some internal validity check to ensure data quality. |

|  |  |
| --- | --- |

**List of abbreviations**

**DALYs**- Disability Adjusted Life Years**, GBD**-Global Burden of Disease, **GSHS**- Global School-based Health Survey, **ICD**- International Classification of Diseases, **MCCD** -Medically Certified Cause of Death, **MORTH**- Ministry of Road Transport and Highways**, NIN**- National Identification Number for Health Facilities, **NSSO** -National Sample Survey Organisation, **NCRB** -National Crimes Record Bureau, **NDSAP** -National Data Sharing Accessibility Policy, **OGD**-Open Government Data**, OPD** - Outpatient Department, **RTA** -Road Traffic Accident**, RTI**- Road Traffic Injury, **SCD** -Survey of Cause of Death, **SRS**- Sample Registration System, **TBSA**-Total Burnt Surface Area, **TRW**-Transport Research Wing, **VA-** Verbal Autopsy, **WHO**-World Health Organisation, **UNICEF** -United Nations International Children’s Emergency Fund

**Supplementary Table 2. National and international data sources of injury morbidity and mortality in India rated according to attributes of injury surveillance**

| Source | Simplicity | Flexibility | Acceptability | Reliability | Utility | Sustainability | Timeliness | Burden | Trends | Risk factors |
| --- | --- | --- | --- | --- | --- | --- | --- | --- | --- | --- |
| ‘Road Traffic’ Injury |  |  |  |  |  |  |  |  |  |  |
| National Crime Research Bureau |  |  |  |  |  |  |  |  |  |  |
| Ministry of Road, Transport and Highways |  |  |  |  |  |  |  |  |  |  |
| Medical Certification Cause of Death |  |  |  |  |  |  |  |  |  |  |
| Cause of Death statistics (SRS) |  |  |  |  |  |  |  |  |  |  |
| National Injury Surveillance Trauma Registry |  |  |  |  |  |  |  |  |  |  |
| National Sample Survey Organisation |  |  |  |  |  |  |  |  |  |  |
| Ministry of Statistics and Program Implementation |  |  |  |  |  |  |  |  |  |  |
| Global status Report on Road Safety |  |  |  |  |  |  |  |  |  |  |
| Open Government Data Platform |  |  |  |  |  |  |  |  |  |  |
| Global Burden Disease Study |  |  |  |  |  |  |  |  |  |  |
| Young Lives  Study |  |  |  |  |  |  |  |  |  |  |
| Source | Simplicity | Flexibility | Acceptability | Reliability | Utility | Sustainability | Timeliness | Burden | Trends | Risk factors |
| ‘Falls’ Injury |  |  |  |  |  |  |  |  |  |  |
| National Crime Research Bureau |  |  |  |  |  |  |  |  |  |  |
| Medical Certification Cause of Death |  |  |  |  |  |  |  |  |  |  |
| National Sample Survey Organisation |  |  |  |  |  |  |  |  |  |  |
| Open Government Data Platform |  |  |  |  |  |  |  |  |  |  |
| Global Burden Disease Study |  |  |  |  |  |  |  |  |  |  |
| Young Lives  Study |  |  |  |  |  |  |  |  |  |  |
| Ministry of Labour and Employment |  |  |  |  |  |  |  |  |  |  |
| National Injury Surveillance Trauma Registry |  |  |  |  |  |  |  |  |  |  |
| Source | Simplicity | Flexibility | Acceptability | Reliability | Utility | Sustainability | Timeliness | Burden | Trends | Risk factors |
| ‘Fire/ Burn’ Injury |  |  |  |  |  |  |  |  |  |  |
| National Crime Research Bureau |  |  |  |  |  |  |  |  |  |  |
| Medical Certification Cause of Death |  |  |  |  |  |  |  |  |  |  |
| National Sample Survey Organisation |  |  |  |  |  |  |  |  |  |  |
| Open Government Data Platform |  |  |  |  |  |  |  |  |  |  |
| Global Burden Disease Study |  |  |  |  |  |  |  |  |  |  |
| Ministry of Labour and Employment |  |  |  |  |  |  |  |  |  |  |
| National Injury Surveillance and Trauma Registry |  |  |  |  |  |  |  |  |  |  |
| Source | Simplicity | Flexibility | Acceptability | Reliability | Utility | Sustainability | Timeliness | Burden | Trends | Risk factors |
| ‘Drowning’ Injury |  |  |  |  |  |  |  |  |  |  |
| National Research Crime Bureau |  |  |  |  |  |  |  |  |  |  |
| Medical Certification Cause of Death |  |  |  |  |  |  |  |  |  |  |
| National Sample Survey Organisation |  |  |  |  |  |  |  |  |  |  |
| Open Government Data Platform |  |  |  |  |  |  |  |  |  |  |
| Global Burden Disease Study |  |  |  |  |  |  |  |  |  |  |
| Ministry of Environment Forest and Climate Change |  |  |  |  |  |  |  |  |  |  |
| Source | Simplicity | Flexibility | Acceptability | Reliability | Utility | Sustainability | Timeliness | Burden | Trends | Risk factors |
| ‘Forces of nature’ injury |  |  |  |  |  |  |  |  |  |  |
| National Research Crime Bureau |  |  |  |  |  |  |  |  |  |  |
| Ministry of Statistics and Programme Implementation |  |  |  |  |  |  |  |  |  |  |
| Global Burden Disease Study |  |  |  |  |  |  |  |  |  |  |
| Source | Simplicity | Flexibility | Acceptability | Reliability | Utility | Sustainability | Timeliness | Burden | Trends | Risk factors |
| ‘Occupational’ Injury |  |  |  |  |  |  |  |  |  |  |
| National Research Crime Bureau |  |  |  |  |  |  |  |  |  |  |
| Open Government Data Platform |  |  |  |  |  |  |  |  |  |  |
| Global Burden Disease Study |  |  |  |  |  |  |  |  |  |  |
| Ministry of Labour and Employment |  |  |  |  |  |  |  |  |  |  |
| Source | Simplicity | Flexibility | Acceptability | Reliability | Utility | Sustainability | Timeliness | Burden | Trends | Risk factors |
| ‘Other’ Injury |  |  |  |  |  |  |  |  |  |  |
| National Sample Survey Organisation |  |  |  |  |  |  |  |  |  |  |
| Integrated disease surveillance system |  |  |  |  |  |  |  |  |  |  |
| National Injury Surveillance and Trauma Registry |  |  |  |  |  |  |  |  |  |  |

**Key: Green= Yes, Red = No, Orange = Somewhat, Yellow = Unsure**

**Supplementary Table 3: Status of core and optional variables collected at data sources**

|  | **DATA AVAILABILITY/DATA SOURCE** | | | | | | | | | | | | |
| --- | --- | --- | --- | --- | --- | --- | --- | --- | --- | --- | --- | --- | --- |
|  | **Pre-hospital** | | | | **Hospital** | | | | | **Police records** | **Insurance** | **Observations** | |
|  | **Call center** | | **Ambulance** | | **Primary** | | **Secondary** | | **Tertiary** |  |  |  | |
| **INDICATOR** | | | | | | | | | | | | | |
| **Core minimum data set (MDS)*** | | | | | | | | | | | | | |
| Identifier (MDS) |  | |  | | x | | x | | x |  | x |  | |
| Age (MDS) |  | | x | | x | | x | | x |  | x | Recorded, not reported in health. Age groups reported in police data | |
| Sex (MDS) |  | | x | | x | | x | | x |  | x | Recorded and reported | |
| Intent (MDS) |  | |  | |  | |  | |  |  | x | Recorded and reported in police but not health | |
| Activity (MDS) |  | |  | |  | |  | |  |  | x |  | |
| Place of occurrence (MDS) | x | | x | |  | | x | | x | x | x | Exact location is noted, however report is based on where case is registered and/or where treated. Police data reports at state, whilst health at district level | |
| Nature of injury (MDS) |  | |  | | x | | x | |  |  |  | Tertiary health facilities used a robust ICD coding system reporting 3 digit  codes using both Chapter XIX and Chapter XX with ICD training | |
| Mechanism of injury (MDS) |  | |  | | v | | v | |  |  |  | Varied from site to site and also from one record to another, at a site. | |
| **Optional data set (ODS)1** | | | | | | | | | | | | | |
| Race/ethnicity (ODS) |  | |  | |  | |  | |  |  |  |  | |
| Date of injury (ODS) |  | | x | |  | | x | | x | x | x | Recorded but reported on monthly basis | |
| Time of injury (ODS) |  | | x | |  | | x | | x | x | x | Reported in class for police report | |
| External cause of injury (ODS) |  | |  | |  | |  | |  | x | x |  | |
| Residence (ODS) |  | | x | |  | | x | |  | x |  |  | |
| Alcohol abuse (ODS) |  | |  | |  | |  | |  | x |  |  | |
| Other psychoactive substance abuse (ODS) |  | |  | |  | |  | |  | x |  |  | |
| Severity (ODS) |  | |  | |  | |  | |  |  |  | Only as major or minor surgeries | |
| Disposition (ODS) |  | |  | | x | | x | | x |  |  |  | |
| Incident summary (ODS) |  | |  | | x | | x | | x | x |  | Usually some history is there but varied from couple words to in-depth information | |
| **Supplementary for traffic injuries** | | | | | | | | | | | | | |
| Mode of transport (MDS) |  | | x | |  | | x | | x | x | x |  | |
| Type of road user (MDS) |  | | x | |  | |  | |  | x | x |  | |
| Counterpart (ODS) |  | |  | |  | |  | |  | x |  |  | |
| **Supplementary for other specific injury** | | | | | | | | | | | | | |
| **Falls** | | | | | | | | | | | | | |
| Type of fall^ |  | |  | | v | | v | | v |  | x | Details for same level fall are less frequently documented as compared to fall from height | |
| Cause of fall^ |  | |  | | v | | v | |  |  | x |  | |
| **Burns/Fire-related** | | | | | | | | | | | | | |
| Type of burn (thermal, chemical, electrical, other) |  |  | |  | | x | | x | | x (if reported) |  | |  |
| Total burn surface area^ |  |  | |  | | x | | x | |  |  | |  |
| First aid provided at home^ |  |  | |  | | v | | v | |  |  | |  |
| **Mechanical** | | | | | | | | | | | | | |
| If animate - animal hit or bitten by^ |  |  | |  | |  | |  | |  |  | |  |
| If inanimate - type of object struck/squashed/hit by^ |  |  | |  | |  | |  | | x | X | |  |
| **Poisoning** | | | | | | | | | | | | | |
| Type of poisoning agent^ |  |  | |  | |  | | x | | x(if reported) |  | |  |
| **Drowning** | | | | | | | | | | | | | |
| Location of drowning (Indoor/outdoor) ^ |  |  | |  | |  | |  | | x |  | | Drowning records were not observed due to low numbers, so details of hospital data are difficult to comment |
| If outdoor, distance from home (metres)^ |  |  | |  | |  | |  | | x |  | |  |
| Type of water body^ |  |  | |  | |  | |  | | x |  | |  |
| **Other Unintentional** | | | | | | | | | | | | | |
| Type of injury |  |  | |  | |  | |  | | x |  | |  |
| **Other demographic indicators** |  |  | |  | |  | |  | |  |  | |  |
| Education^ |  |  | |  | |  | |  | |  |  | |  |
| Marital status^ |  |  | |  | |  | |  | | x | x | |  |
| Occupation^ |  |  | |  | |  | | x | | x | v | |  |
| Type of insurance^ |  |  | |  | |  | |  | |  | x | |  |
| **Other injury-related indicators** |  |  | |  | |  | |  | |  |  | |  |
| Injury severity+ |  |  | |  | |  | |  | |  |  | | Some classification is expected for insurance data, however it relied on medical records and no severity coding or classification is used |
| Part of body injured^ |  |  | | x | | x | | x | |  | x | |  |
| **Diagnosis & referral** | | | | | | | | | | | | | |
| First aid given before reaching hospital^ |  |  | | v | | v | | x | |  |  | |  |
| Date of presentation^ | x |  | |  | | x | | x | |  |  | |  |
| Time of presentation^ | x |  | |  | | x | | x | |  |  | |  |
| Patient accompanied by^ |  |  | | x | | x | | x | |  |  | |  |
| Date of discharge^ |  |  | | x | | x | | x | |  |  | |  |
| Time of discharge^ |  |  | | x | | x | | x | |  |  | |  |
| Case referred^ |  |  | | x | | x | | x | |  |  | |  |
| Clinical diagnosis^ |  |  | | x | | x | | x | |  |  | |  |
| ICD Classification^ |  |  | |  | | x | | x | |  |  | | As in text, secondary facilities group the codes |
| AIS score^ |  |  | |  | |  | |  | |  |  | |  |
| Co-morbidities^ |  |  | | x | | x | | x | |  |  | |  |
| Discharge summary^ |  |  | | x | | x | | x | |  |  | |  |
| Investigations^ |  |  | |  | | x | | x | |  |  | |  |
| **Outcome** |  |  | |  | |  | |  | |  |  | |  |
| Treatment^ |  |  | | x | | x | | x | | x |  | |  |
| Outcome^ |  |  | | x | | x | | x | | x |  | |  |
| Date of death |  |  | | x | | x | | x | | x |  | |  |
| Time of death |  |  | | x | | x | | x | | x |  | |  |

Key:

x – Information collected, as observed

v- Variable, data collection is heterogeneous

*These 8 variables are the recommended basic international standard data set for comparison (core MDS) against countries from WHO Injury Guidelines

+ Optional data set (ODS) from WHO Injury Guidelines

^ These variables were additional from Appendix of the WHO Injury Surveillance Proposal and the National Injury Surveillance Centre Trauma data capture form

**Appendix 1: Adapted WHO injury surveillance evaluation tool**

We adapted the WHO injury surveillance evaluation tools for this work, using online data collection using Research Electronic Data Capture (REDCAP. However, data collection on a closed structure form limited our capacity to identify inefficiencies and to explore bigger issues around resources, administration, reporting and the like. Additionally, there were no directives/guidelines for the health facilities to be accountable for with respect to injury data. We therefore also took a more explorative approach and identified the following through the site visits:

- - 1. What is the population under surveillance? Or who is being represented in the data source?
    2. What is the period of time of the data collection?
    3. What information is collected? Injury details, causes, sociodemographic and like
    4. Who provides the injury data information?
    5. How is the information transferred?
    6. How is the information stored?
    7. Who compiles, analyses or reports the data?
    8. How are the data analysed and how often?
    9. How often are reports disseminated?
    10. To whom are reports distributed?

The selection of evaluation sites was guided by identification of areas with high injury rates reported by the National Crime Record Bureau, known high risk areas for specific injuries such as drowning in the north east, geographic representation, and collaborative links guided the selection of evaluation sites. Sub centres (which do not attend to injuries), primary health care centres, district hospitals, tertiary care centres, private and charitable hospitals, ambulance posts, police posts, and insurance offices were visited. Interviews were conducted with a range of relevant personnel such as emergency registration clerk, information assistant, nurses, medical officer, civil surgeon, medical record officer, ambulance drivers, attendants, call centre staff, insurance officers and police officials. Data were collected between January – March 2017.

For the state of Himachal Pradesh, Haryana, Punjab, Gujrat, Telangana and Uttar Pradesh health facilities from Primary Health Centres (PHCs), Community Health Centres (CHCs) and District hospitals were visited. Tertiary centres were visited in Chandigarh, Gujrat, and Tamil Nadu. Private hospitals in Panchkula, Haryana and Pune, Maharashtra were covered. Interviews were conducted with staff who attended the injured person at point of entry to the health facility and anyone collecting information on patient history, to medical records department/administrative personnel responsible for record keeping and reporting.
